# Acupuncture gone awry: surgical management of a popliteal artery pseudoaneurysm with concurrent abdominal aortic aneurysm

**DOI:** 10.1093/jscr/rjae837

**Published:** 2025-01-08

**Authors:** Sebastian Asteguieta, Carlos Diaz Q, Luis P Jacobs, Kelly Villeda, Sebastian Estrada

**Affiliations:** Department of Research, Universidad Francisco Marroquín, Guatemala City, Guatemala; Department of Research, Universidad Francisco Marroquín, Guatemala City, Guatemala; Department of Research, Universidad Francisco Marroquín, Guatemala City, Guatemala; Department of Research, Universidad Francisco Marroquín, Guatemala City, Guatemala; Department of Research, Universidad Francisco Marroquín, Guatemala City, Guatemala

**Keywords:** popliteal pseudoaneurysm, aortic aneurysm, elderly patient, multidisciplinary care, comorbidities

## Abstract

Popliteal artery pseudoaneurysms are rare, especially from acupuncture-related trauma. We report a 67-year-old male with hypertension, diabetes, chronic kidney disease, and an abdominal aortic aneurysm (AAA), who developed a popliteal pseudoaneurysm after acupuncture. Imaging confirmed the pseudoaneurysm and a 55 mm AAA. Urgent surgical repair with a Gore-Tex graft led to a smooth recovery. This case emphasizes the vascular risks of acupuncture and the importance of early diagnosis, multidisciplinary care, and personalized follow-up in patients with complex comorbidities.

## Introduction

Pseudoaneurysms of the popliteal artery are notably uncommon, primarily associated with penetrating trauma rather than blunt trauma. The incidence, such cases are even rarer, often linked to iatrogenic causes (such as surgical interventions) or traumatic injuries like fractures or vascular access procedures. Their rarity makes diagnosis challenging, and untreated cases can lead to severe complications, including thrombosis or limb ischemia. Timely identification and intervention are crucial to prevent further vascular compromise and ensure optimal patient outcomes, these pseudoaneurysms account for only 0%–3.5% of all popliteal aneurysms [1, 2]. The incidence of pseudoaneurysms varies widely, ranging from 0.1% to 6% and even up to 0.5%–9%, depending on the diagnostic or therapeutic procedure involved [3].

A pseudoaneurysm is a rare vascular condition where blood escapes through a disrupted arterial wall, forming a hematoma encapsulated by external tissue layers. In this case, a 67-year-old male developed a popliteal pseudoaneurysm following acupuncture therapy. Complicating his history, the patient also has an abdominal aortic aneurysm (AAA), hypertension, diabetes, and chronic kidney disease. The pseudoaneurysm was treated with surgical resection and a Gore-Tex graft. Despite his complex medical profile, the patient recovered smoothly, with stable vital signs and no respiratory complications. He remains under multidisciplinary care for future repair of the abdominal aneurysm [4].

## Case presentation

A 67-year-old male presented with progressive swelling, tenderness, and discoloration in the back of his right knee, which began three days after an acupuncture session to relieve chronic knee pain. Initially, the discomfort was mild and attributed to post-treatment soreness, but it worsened, accompanied by pulsating sensations, bruising, and swelling, prompting him to seek care.

**Figure 1:**
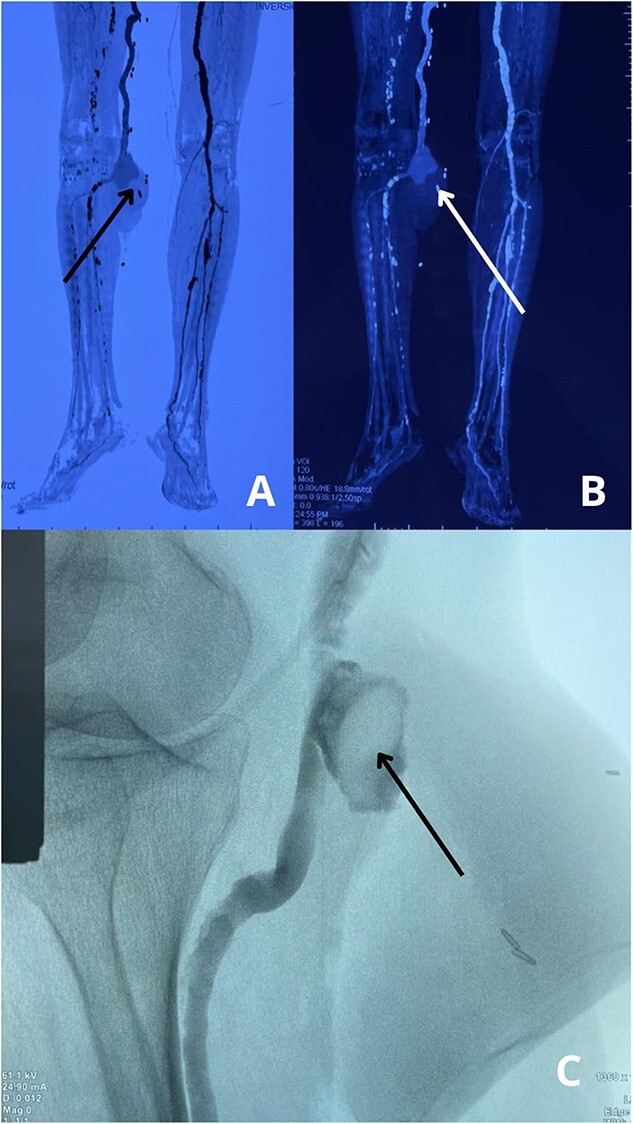
(A) Preoperative view of the right leg, showing swelling, bruising, and discoloration at the site of the popliteal pseudoaneurysm. (B) Intraoperative view displaying the placement of a Gore-Tex graft to restore arterial flow after excision of the pseudoaneurysm.

His medical history included a diagnosed AAA 2 years earlier, monitored for surgical repair, along with hypertension managed with amlodipine and type 2 diabetes mellitus controlled with metformin. During the recent acupuncture session, needles were placed in the popliteal fossa, an area with dense neurovascular structures, including the popliteal artery. On examination, the patient was hemodynamically stable with normal vital signs. The right knee showed swelling, ecchymosis, tenderness, and a pulsatile mass in the popliteal fossa, suggesting vascular injury. Peripheral pulses were diminished, but sensory and motor functions remained intact (Fig. 1A).

**Figure 2:**
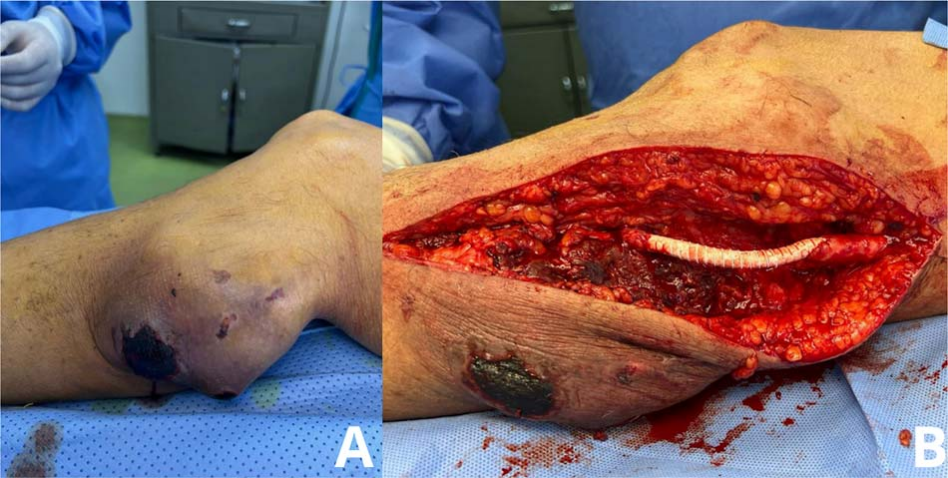
(A, B) CTA scans of the lower extremities reveal a pseudoaneurysm in the right popliteal artery, indicating vascular injury with disrupted arterial flow. (C) Angiographic study shows the pseudoaneurysm sac and confirms a tear in the popliteal artery, with no evidence of distal thrombus or embolization.

An ultrasound Doppler revealed turbulent blood flow within a pseudoaneurysm from the popliteal artery. A computed tomography angiogram (CTA) confirmed a 45 × 40 mm pseudoaneurysm with a small arterial tear and a surrounding hematoma compressing adjacent structures (Fig. 2A–C). The CTA also updated the size of the AAA, now 55 mm, requiring careful planning for surgical intervention to prevent rupture while addressing both vascular issues.

**Figure 3:**
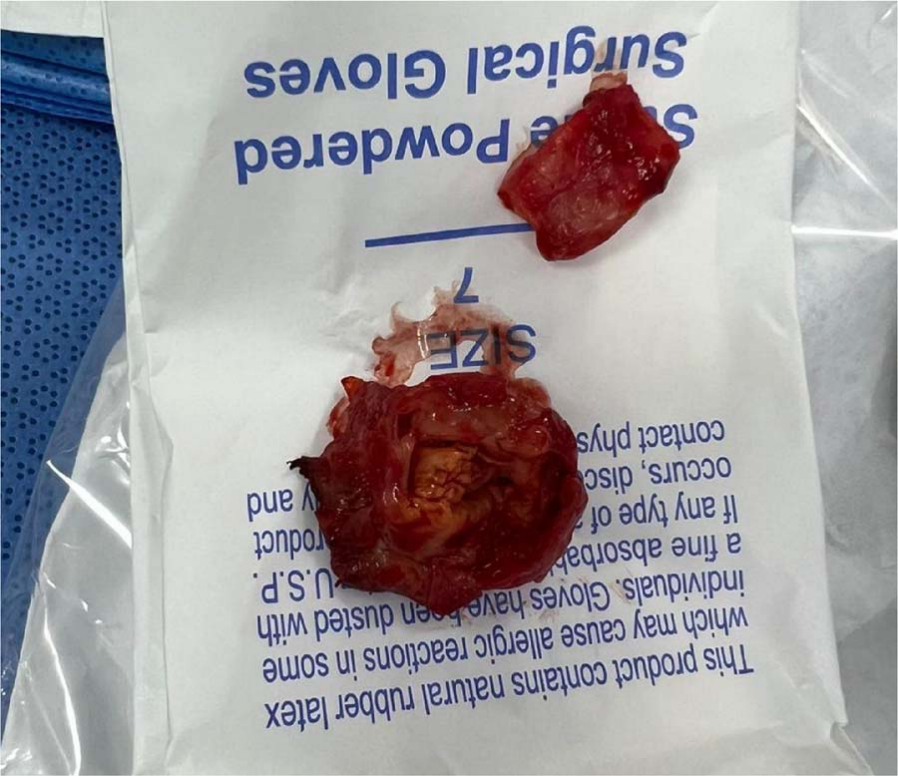
The image shows the excised pseudoaneurysm from the popliteal artery, obtained during surgical intervention. The specimen reveals thickened arterial walls and organized hematoma, indicative of chronic vascular injury.

The patient underwent urgent surgical resection of the popliteal pseudoaneurysm with the placement of a 10 cm Gore-Tex graft (Fig. 1B and 3), confirming intraoperatively a 5 mm arterial tear likely caused by a misplaced acupuncture needle, with no distal thrombus or emboli detected. He was transferred to the ICU for monitoring, where recovery was complicated by mild hyperlactatemia, but respiratory function stabilized, allowing discontinuation of oxygen therapy within 24 h. Blood glucose was managed with insulin to promote wound healing and prevent infection.

The patient was discharged on postoperative day seven with instructions for wound care, glucose monitoring, follow-up, and a recommendation to avoid acupuncture in high-risk areas.

## Discussion

This case highlights the rare occurrence of a pseudoaneurysm caused by acupuncture therapy, illustrating the potential complications associated with alternative treatments. While acupuncture is generally considered safe, improper needle placement in anatomically sensitive regions, such as the popliteal fossa, can lead to severe vascular injuries [5, 6]. Pseudoaneurysms result from a tear in the arterial wall, allowing blood to collect between tissue layers [7]. In this case, the injury caused by the acupuncture needle created a pseudoaneurysm that required urgent surgical intervention.

The management of this patient also underscores the complex interplay of multiple vascular conditions. The presence of both a popliteal pseudoaneurysm and an AAA required careful planning to prioritize surgical interventions [8]. The decision to address the pseudoaneurysm first reflects the immediate risk of rupture and embolization, while the abdominal aneurysm repair was deferred to allow for recovery from the initial procedure.

The use of a Gore-Tex graft provided long-term durability and minimized the risk of future rupture, although postoperative surveillance remains critical to detect potential complications [9]. While endovascular repair is often preferred in elderly patients with multiple comorbidities, the need for direct arterial repair following needle trauma made open surgery the best option in this case.

The gold standard for diagnosing pseudoaneurysms is computed tomography angiography due to its high accuracy in assessing vascular injuries [10]. Treatment options include endovascular repair or open surgical resection with graft placement [11]. In this patient, surgical resection was preferred due to the size of the pseudoaneurysm, arterial tear, and the need for direct repair, highlighting the importance of multidisciplinary management to ensure optimal recovery and prevent recurrence.

## Conclusion

This case highlights the rare occurrence of a popliteal artery pseudoaneurysm secondary to acupuncture-related trauma, emphasizing the importance of rapid diagnosis to prevent complications such as rupture or embolization. Open surgical repair with Gore-Tex graft placement was essential due to the arterial injury. The presence of a concurrent AAA required strategic planning, deferring elective repair until recovery from the initial intervention. Multidisciplinary management was critical to address comorbidities, ensuring optimal postoperative outcomes and minimizing risks associated with the patient’s complex vascular profile.

## Data Availability

All data supporting this case report are included in the article, with no additional data available due to patient confidentiality.
